# Effects of temperature frequency trends on projected japonica rice (*Oryza sativa* L.) yield and dry matter distribution with elevated carbon dioxide

**DOI:** 10.7717/peerj.11027

**Published:** 2021-03-11

**Authors:** Zeyu Zhou, Jiming Jin, Libing Song, Ling Yan

**Affiliations:** 1 College of Water Resources and Architectural Engineering, Northwest A&F University, Yangling, Shaanxi Province, China; 2 Key Laboratory of Agricultural Soil and Water Engineering in Arid and Semiarid Areas, Ministry of Education, Northwest A&F University, Yangling, Shaanxi Province, China

**Keywords:** CERES-Rice model, Temperature frequency, Photosynthetic production, CO_2_, Fertilized spikelet number, Rice yield

## Abstract

In this study, we investigated the effects of temperature frequency trends on the projected yield and dry matter distribution of japonica rice (*Oryza sativa* L.) with elevated carbon dioxide (CO_2_) under future climate change scenarios in northwestern China. The Crop Environment Resource Synthesis (CERES)-Rice model was forced with the outputs from three general circulation models (GCMs) to project the rice growth and yield. Future temperature trends had the most significant impact on rice growth, and the frequency of higher than optimal temperatures (∼24–28 ^o^C) for rice growth showed a marked increase in the future, which greatly restricted photosynthesis. The frequency of extreme temperatures (>35 ^o^C) also increased, exerting a strong impact on rice fertilization and producing a significantly reduced yield. Although the increased temperature suppressed photosynthetic production, the elevated CO_2_ stimulated this production; therefore, the net result was determined by the dominant process. The aboveground biomass at harvest trended downward when temperature became the major factor in photosynthetic production and trended upward when CO_2_-fertilization dominated the process. The trends for the leaf and stem dry matter at harvest were affected not only by changes in photosynthesis but also by the dry matter distribution to the panicles. The trends for the rice panicle dry matter at harvest were closely related to the effects of temperature and CO_2_ on photosynthetic production, and extreme temperatures also remarkably affected these trends by reducing the number of fertilized spikelets. The trends of rice yield were very similar to those of panicle dry matter because the panicle dry matter is mostly composed of grain weight (yield). This study provides a better understanding of the japonica rice processes, particularly under extreme climate scenarios, which will likely become more frequent in the future.

## Introduction

Rice (*Oryza sativa* L.) is one of the most important grain crops to ensure current and future food security. Due to its high suitability for many growing environments, rice can be planted in a wide range of climate conditions (Maclean et al., 2002). Except for Antarctica, rice is grown on all continents, with a planting area of more than 1.6 million km^2^ (FAOSTAT, 2017). With such a vast area, rice has become the staple food for over half the world’s population (Gross & Zhao, 2014; Sawano et al., 2008). However, Wailes & Chavez (2012) predicted that rice consumption would continue to increase with increasing population but that the planting area would remain unchanged. Therefore, high rice production is essential for food security and even social stability.

Compared with the rice grown in low-latitude regions (indica rice), japonica rice planted in mid-latitude regions has a higher yield. Generally, japonica rice has a longer growth period than indica rice, resulting in a longer grain filling duration and thus a higher yield (Ying et al., 1998; Kropff et al., 1994). In addition, the sunshine hours in mid-latitude regions are longer than those in low-latitude regions during the summer (the rice growth period). For example, in China, the sunshine hours in Jilin, Liaoning, and Ningxia provinces (mid-latitude regions) are ∼7.4–8.4 h during the rice growth period over 1960 through 2014, but are ∼5.9–6.0 h in Fujian and Guangdong provinces (low-latitudes regions) (these data were provided by the China Meteorological Administration). Such longer sunshine hours are beneficial for the higher photosynthetic production (Yu et al., 2014; Zhang et al., 1992) of japonica rice.

Although the cultivars, management practices, rice types or other factors are associated with the rice yield, the sunshine hours still play a significant role. In China, rice yields in most low-latitude provinces were less than 6,000 kg/ha, but they were over 8,000 kg/ha in the mid-latitude provinces (National Bureau of Statistics of China, 2007-2016). The average indica rice yields were only about 2,200 kg/ha in India, a low-latitude country (Ministry of Agriculture in India, 2015). Japonica rice yields reached 7,000–8,000 kg/ha in Japan (Kato, Okami & Katsura, 2009), and even 10,700 kg/ha in Australia (Chauhan, Jabran & Mahajan, 2017), both of which are mid-latitude countries. Therefore, the high yield of japonica rice is an important reason for it to be planted in the mid-latitudes.

Climate change has significant effects on rice growth and yield. When the daytime temperature (DT) is above the optimal value (∼26 °C) for rice growth (Singh & Padilla, 1995), photosynthesis is weakened (Mathur, Agrawal & Jajoo, 2014; Feng et al., 2007; Makino & Sage, 2007) and grain filling duration is shortened (Abayawickrama et al., 2017; Ahmed et al., 2015; Kim et al., 2011b), resulting in a decreased yield. When the daily maximum temperature is over 35 °C, rice spikelet fertilization is greatly restricted (Madan et al., 2012; Jagadish, Craufurd & Wheeler, 2007; Jagadish, Craufurd & Wheeler, 2008; Yoshida, Satake & Mackill, 1981; Satake & Yoshida, 1978), further lowering the yield. On the other hand, carbon dioxide (CO_2_) is critical for photosynthesis, and elevated CO_2_ concentrations can accelerate carbon assimilation, enhancing photosynthetic production and increasing the rice yield (Yang et al., 2006; Lieffering et al., 2004; Kimball, Kobayashi & Bindi, 2002). Hence, examining the combined effects of temperature and CO_2_ is essential to the prediction of rice production under climate change.

Field experiments in chambers have been conducted with different combinations of temperature and CO_2_ concentration. Roy et al. (2012) carried out a field experiment in India for three years to observe the effects of ambient CO_2_ (390 ppm in a control chamber), elevated CO_2_ (550 ppm), and elevated CO_2_ (550 ppm) with elevated temperature (+2 °C over the control chamber) on dry matter production, and the CO_2_ treatments were maintained throughout the entire rice growth season. Compared with the control chamber, the photosynthesis and each rice organ dry matter had a significant increase under the other two treatments, and there were no significant differences between these two treatments.

Kim et al. (2011a) also conducted a similar experiment in South Korea, with 380 ppm ambient CO_2_, 662 ppm elevated CO_2_, and 2 °C elevated temperature. Their results suggested that rice photosynthesis was enhanced with elevated CO_2_ and/or temperature; however, the whole plant dry matter in elevated CO_2_ and temperature was much larger than that in elevated CO_2_ alone. In addition, the dry matter distribution showed an inconsistent trend in different organs. The elevated temperature effect reduced the dry matter allocation to the panicle due to decreased spikelet numbers, significantly increasing the leaf and stem dry matter, while the elevated CO_2_ can alleviate this effect. Thus, the panicle dry matter under elevated CO_2_ and temperature was less than that in elevated CO_2_; however, the dry matter of the leaf and stem was larger than the latter.

Although the experimental operation matters, the different magnitude combinations of temperature and CO_2_ concentration set were a key reason causing the differences between their results. This also resulted in differences in the dry matter accumulation and distribution among rice organs in recent studies (Wang et al., 2019a; Nam et al., 2013; Cheng et al., 2010). The temperature and CO_2_ concentration are closely coupled at a long-term temporal scale, and the combinations of these two variables in the experimental chambers may not reflect the actual long-term relationship between them. Hence, the response of rice growth to temperature and CO_2_ concentration may be different in reality.

Crop models are driven by various combinations of temperature and CO_2_ concentration generated with general circulation models (GCMs) (Kim et al., 2013; Iizumi, Yokozawa & Nishimori, 2011; Tao et al., 2008). These combinations are based on the physical relationship between these two variables, overcoming the drawback of the often randomly set values in the experimental chambers. In addition, these modeling studies indicated that high temperature shortened the rice growth period and reduced the yield, while CO_2_-fertilization tended to increase the yield (Kim et al., 2013; Iizumi, Yokozawa & Nishimori, 2011; Tao et al., 2008). However, less attention has been paid to changes in the physiological processes during rice growth with different combinations of temperature and CO_2_ concentration, which needs to be further investigated.

The objectives of this study are: (1) to quantify the effects of different combinations of temperature and CO_2_ concentration on the japonica rice yield and dry matter distribution with the Crop Environment Resource Synthesis (CERES)-Rice model driven by the outputs of three GCMs with different greenhouse gas emission scenarios, and (2) to explore the influence of extreme temperature (>35 °C) frequency projections on japonica rice growth and yield. In the following, ‘Materials & Methods’ introduced the study area, data, and methodology, ‘Results’ presents the results, ‘Discussion’ includes the discussion, and ‘Conclusion’ describes the conclusions.

## Materials & Methods

### Study area

**Figure 1:**
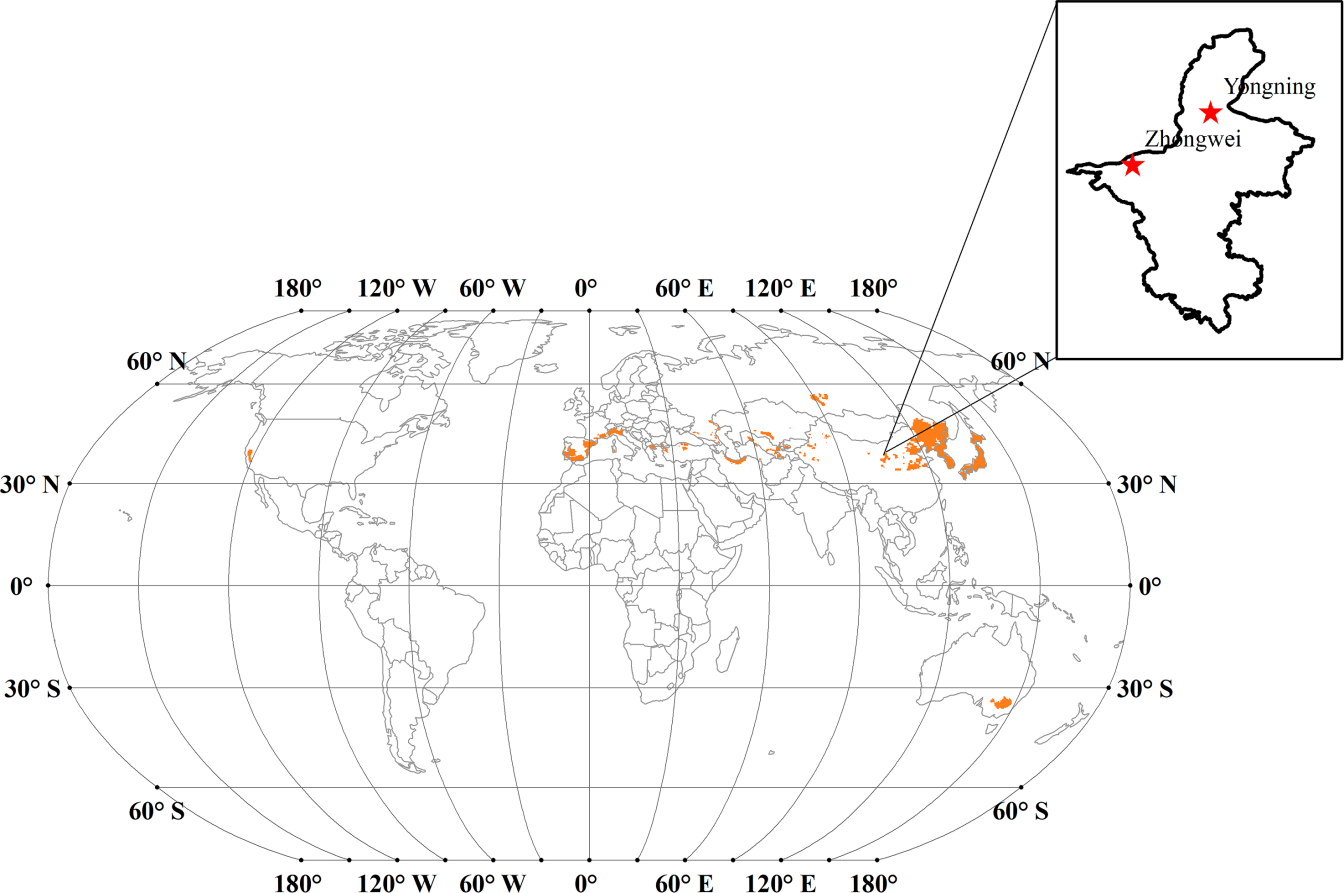
The major japonica rice cultivation areas (orange areas) across the world. Note: Map of rice production across the world is from Andrew (2010), and the license of this map is CC BY SA 3.0. Available at: https://commons.wikimedia.org/w/index.php?curid=9643470 We extracted the primary japonica rice planting regions based on Chauhan, Jabran & Mahajan (2017) and Thompson (2002).

Japonica rice is typically grown in temperate regions (Chauhan, Jabran & Mahajan, 2017; Mackill & Lei, 1997). The major japonica rice cultivation areas are located in northeastern China, Korea, Japan, New South Wales of Australia, and European countries among the Mediterranean Sea (Fig. 1). We calculated the annual mean cumulative growing degree days (GDD) over 1901 through 2016 for these regions based on the temperature data from the Climate Research Unit-National Centers for Environmental Prediction (CRUNCEP; Viovy, 2011). The cumulative GDDs were mostly between 1,000–2,600  °C day in these regions, and the base temperature was set to 9 °C in our calculation based on Singh, Ritchie & Godwin (1993). For this study, we selected the Zhongwei (37.53°N, 105.18°E) and Yongning (38.25°N, 106.25°E) rice field stations, which are located about 125 km apart in Ningxia province, China (Fig. 1) and have a typical temperate continental climate. Due to the high production and excellent quality, japonica rice has been planted in this region for about 2,000 years (Wang et al., 2013) where the GDD is about 1,800 °C day based on the temperature data for the period of 1901–2016.

### Input data for the CERES-Rice model

Historical weather data, field management information, soil properties, and rice cultivar parameters are needed to drive the CERES-Rice model. Historical weather data were derived from the China Meteorological Forcing Dataset (CMFD) (He et al., 2020; Yang et al., 2010), including the daily maximum temperature (T_max_), daily minimum temperature (T_min_), daily precipitation, and daily incoming solar radiation. These data cover China with a spatial resolution of 0.1° at 3-hour time steps for the period of 1979 to 2015. In this study, we averaged the weather data for each station over the nine surrounding grids to reduce the uncertainty. The field management information required by the CERES-Rice model was provided by the Ningxia Meteorological Bureau, China. This information included detailed phenology dates, yields, amounts and dates of fertilization and irrigation, etc. Soil data were collected from the China soil database (http://vdb3.soil.csdb.cn/) as shown in Table 1. We used the rice cultivar Ningjing No. 16, a common japonica rice cultivar in the study area. All data from the Zhongwei station were used to calibrate the CERES-Rice model, and data from the Yongning station were used for model validation.

**Table 1:** Detailed information about soil properties.

Depth (cm)	Clay %	Silt %	Sand %	Organic carbon (g/kg)	Total nitrogen (g/kg)	pH	Cation exchange capacity (cmol/kg)
0–20	23.0	33.7	43.3	14.8	1.10	8.1	11.1
20–55	25.0	32.5	42.5	11.2	1.07	8.4	10.3
55–120	18.5	31.5	50.0	7.0	0.43	8.4	9.4

The future climate data used to drive the CERES-Rice model were derived from the outputs of three GCMs (Table 2) with four Representative Concentration Pathways (RCPs) (2.6, 4.5, 6.0, and 8.5) from the Coupled Model Intercomparison Project Phase 5 (Taylor, Stouffer & Meehl, 2012). These three GCMs represent different warming intensities, where the temperature increase was the most aggressive in IPSL-CM5A-MR, moderately aggressive in MIROC5, and least aggressive in GFDL-ESM2G (Hsu et al., 2013). The variables we used from these GCMs were T_max_, T_min_, precipitation, and incoming solar radiation. We also used the projected future CO_2_ concentrations from Prather et al. (2013) to consider the effects of CO_2_ on rice growth. Future projections in this study were made using the cultivar Ningjing No. 16, and the transplanting date was set to May 12 each year, a time when rice transplanting is commonly done (Liu et al., 2017; Sang, Liu & Qiu, 2006). According to the local farmers’ experience, transplanting was set at a hill spacing of 25 ×10 cm with six seedlings per hill (Chen, 2012; Ma et al., 2003; Wang, Yin & An, 1996). A total of 290 kg/ha of nitrogen fertilizer was applied to the rice field each year, the maximum value in the records, to ensure that there was no nitrogen stress during the rice growth period. The other field management information, such as full irrigation, and soil data were the same as in the historical runs.

**Table 2:** Detailed information about the three GCMs selected in this study.

GCM	Resolution	Modeling Center
MIROC5	1.4° × 1.4°	Atmosphere and Ocean Research Institute (The University of Tokyo), National Institute for Environmental Studies, and Japan Agency for Marine-Earth Science and Technology
IPSL-CM5A-MR	2.5° × 1.3°	Institute Pierre-Simon Laplace MR: Medium resolution
GFDL-ESM2G	2.5° × 2.0°	Geophysical Fluid Dynamics Laboratory

### Methodology

#### The CERES-Rice model

The CERES-Rice model is one of the crop models in the Decision Support System for Agrotechnology Transfer (DSSAT) family (Version 4.6) (Jones et al., 2003). The model simulates rice phenology development based on the GDDs calculated with the daily average temperature. Photosynthetic production accumulation and allocation to each organ are associated with the growth stage and the stress factors, such as temperature, CO_2_, nitrogen, and water. In this study, no nitrogen or water stress existed during the rice growth period, and temperature and CO_2_ were the main factors affecting photosynthesis. In the CERES-Rice model, the optimal DT for rice photosynthesis is set at 26 ° C (Singh & Padilla, 1995). A quadratic equation is used to calculate the temperature reduction factor (PRFT) that affects photosynthesis (Ritchie et al., 1998):

(1) \begin{eqnarray*}PRFT=1.0-0.0025\times ((0.75{T}_{\mathrm{max}}+0.25{T}_{\mathrm{min}})-26.0)^{2}\end{eqnarray*}

PRFT is applied to the calculation of photosynthetic production in the CERES-Rice model. When PRFT is equal to 1.0, this indicates no temperature stress on photosynthesis, and when it is equal to 0.0, there is the maximum temperature stress. The effects of CO_2_ concentration on rice photosynthesis are also taken into account in the CERES-Rice model (Peart et al., 1988). A factor (PCO2) representing these effects was applied to the calculation of photosynthetic production based on Table 3. In this lookup table, the PCO2 value and corresponding CO_2_ concentration are prescribed in the model based on Peart et al. (1988). The PCO2 value of any given CO_2_ concentration was calculated with a linear interpolation between the two nearest factors whose weights were computed according to the given and two corresponding CO_2_ concentrations.

**Table 3:** Effect of CO_2_ on rice photosynthesis.

CO_2_/ppm	0	220	330	440	550	660	770	880	990	9999
PCO2	0.00	0.71	1.00	1.08	1.17	1.25	1.32	1.38	1.43	1.50

#### Downscaling and trend calculation

For this study, statistical downscaling was performed to transform the coarse-resolution GCM data (1.3°–2.5°) into the station data from Zhongwei and Yongning to reduce the uncertainty in the GCM data. The CMFD data were used as observations for downscaling. We first used bilinear interpolation to downscale these 3-hourly GCM data, except precipitation, to the resolution of CMFD (0.1°). Second, the downscaled data were statistically bias-corrected by maintaining the probability distributions of historical values similar to those of CMFD through linear regression models (Dettinger et al., 2004). In addition, we used daily downscaled precipitation data (Abatzoglou, 2013) with the method described by Buotte et al. (2019) to create 3-hourly precipitation data for the selected GCMs, and then interpolated these new precipitation data to 0.1° using bilinear interpolation. Through downscaling and bias-corrections, the biases in these GCM outputs were reduced for the historical period, making the future projections more reliable. Meanwhile, the long-term trends of the downscaled climate and simulated rice growth and development variables were calculated for the period of 2006–2100. The significance of these trends was examined with the Mann–Kendall test (Hamed, 2008) using R programming language.

**Figure 2:**
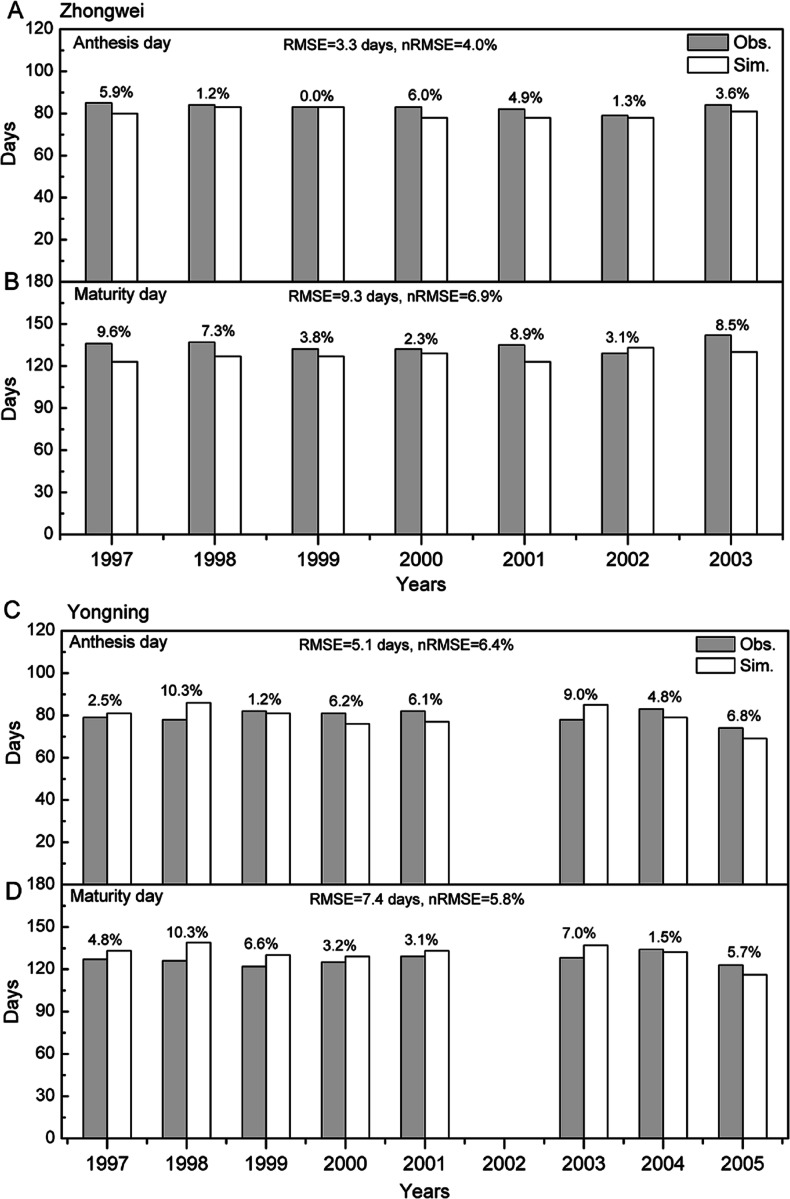
Comparison between rice phenology simulations and observations for (A –B) the Zhongwei station and (C–D) the Yongning station. The numbers above the bars are AREs. RMSE is root mean square error, and nRMSE is equal to RMSE divided by the mean value of observations.

## Results

### Parameter estimation and model evaluation

**Figure 3:**
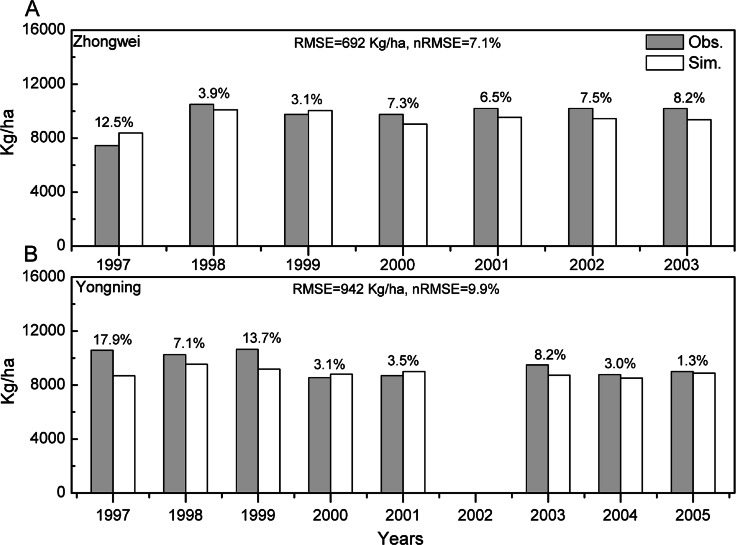
Comparison between rice yield simulations and observations for (A) the Zhongwei station and (B) the Yongning station. The numbers above the bars are AREs. RMSE is root mean square error, and nRMSE is equal to RMSE divided by the mean value of observations.

The crop genotype parameters in CERES-Rice were optimized with the Generalized Likelihood Uncertainty Estimation (GLUE) package (He et al., 2010) using seven-year available agricultural experiment data from the Zhongwei station. These optimized parameters were validated with data from the Yongning station (Table 4). In this study, we used the absolute relative error (ARE) and the least normalized root mean square error (nRMSE) to evaluate the performance of the CERES-Rice model. The largest AREs of the anthesis and maturity dates were less than 11% for the two stations. Except for the Yongning station in 1997, all the AREs of the simulated rice yield were less than 13% (Figs. 2 and 3). Ritchie et al. (1998) indicated that the quality of DSSAT simulations with AREs of less than 15% are considered acceptable. In addition, He et al. (2017) suggested that a crop model performance is considered reasonable when the nRMSE is less than 15%. Therefore, the GLUE-optimized genotype parameters for the CERES-Rice model can be employed to study the effects of future climate on rice growth and development.

**Table 4:** Genotype parameters of Ningjing No. 16 for the CERES-Rice model.

Genotype parameter	GLUE Optimized value
P1: GDD for basic vegetative phase (^∘^C day)	321.9
P2O: Critical photoperiod (hour)	12.5
P2R: Photoperiod sensitivity coefficient (^∘^C day)	51.5
P5: GDD from beginning of grain filling to maturity (^∘^C day)	508.0
G1: Potential grain number coefficient	55.7
G2: Single grain weight (g)	0.024
G3: Tillering coefficient	1.0
G4: Temperature tolerance coefficient	1.0
PHINT: phyllochron	83.0

### Climate change projections

#### GCM data downscaling

**Figure 4:**
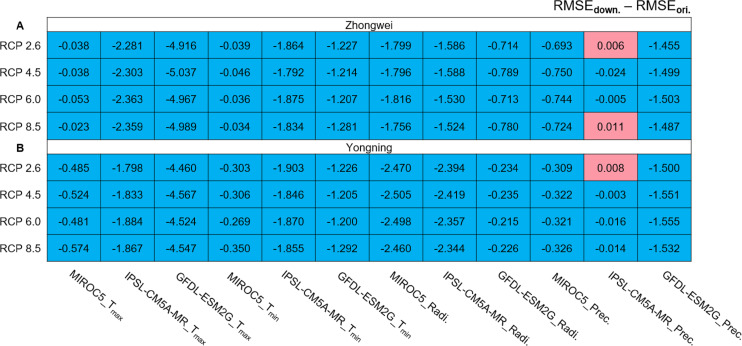
RMSE differences between downscaled and original GCM variables during 1979–2005 for (A) the Zhongwei station and (B) the Yongning station. For T_max_ and T_min_ the unit is ° C, for radiation it is MJ/m^2^, and for precipitation it is mm.

We calculated the root mean square error (RMSE) differences between the downscaled and original GCM data for the period of 1979–2015 (Fig. 4). The observational data used for the calculations were the CMFD data. All the RMSEs were reduced with the downscaled data except for those with precipitation in the three red boxes simulated by IPSL-CM5A-MR (Fig. 4). These RMSE increases were very minor and negligible. Thus, statistical downscaling improved the quality of the historical GCM data and should be capable of generating more reliable future projections.

#### Trends of climate variable projections

**Figure 5:**
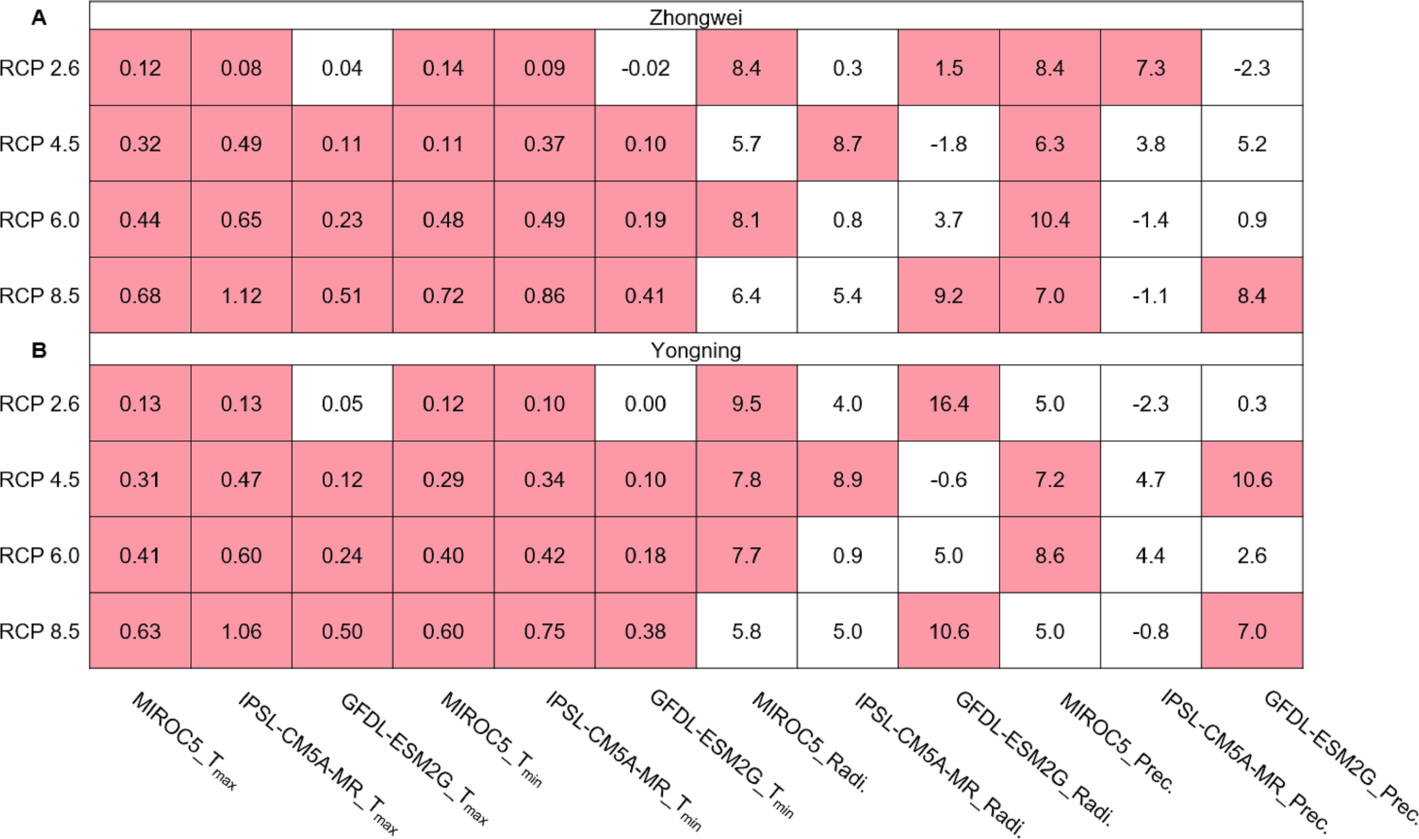
Trends of T_max_ and T_min_ (°C/decade), solar radiation (MJ/decade), and precipitation (mm/decade) during the rice growth period over 2006–2100 for (A) the Zhongwei station and (B) the Yongning station. The red boxes show upward trends that pass the significance level test (*p* < 0.05), and the white boxes mean that this test is not passed.

The annual averaged T_max_ and T_min_ showed a significantly upward trend with most greenhouse gas emission scenarios over the rice growth period, while the solar radiation and precipitation trends were mostly insignificant over the same period (Fig. 5). In most of our study cases (92%), the trends of T_max_ and T_min_ passed the 95% significance level (*p* < 0.05). The significant trend for T_max_ ranged from 0.08 to 1.12 °C /decade, while it ranged from 0.09 to 0.86 °C /decade for T_min_. The largest T_max_ and T_min_ trends occurred with IPSL-CM5A-MR for RCP 8.5. Thus, these significant trends may affect future rice growth and development projections, as analyzed in the following sections.

#### Trends of temperature frequency

In the CERES-Rice model, DT is defined as 0.75T_max_ + 0.25T_min_ (Ritchie et al., 1998), a temperature that is essential for photosynthesis. For this study, we divided the DT into four intervals: <24  °C, (24 °C, 28 °C], (28 °C, 35 °C], and >35  °C. The CERES-Rice model sets 26 °C as the optimal DT for photosynthesis (Singh & Padilla, 1995), and DTs within the range of (24 °C, 28  °C] reduce rice photosynthesis by less than 1% based on Eq. (1), which is negligible. Hence, for rice photosynthesis, DTs falling within this temperature range were defined as optimal (OT), <24 °C were the low temperature (LT), (28  °C, 35 °C] were the high temperature (HT), and >35 °C were the extreme temperature (EMT). When the DT was outside the optimal range in the CERES-Rice model, this played a significant role in restricting rice photosynthesis.

**Figure 6:**
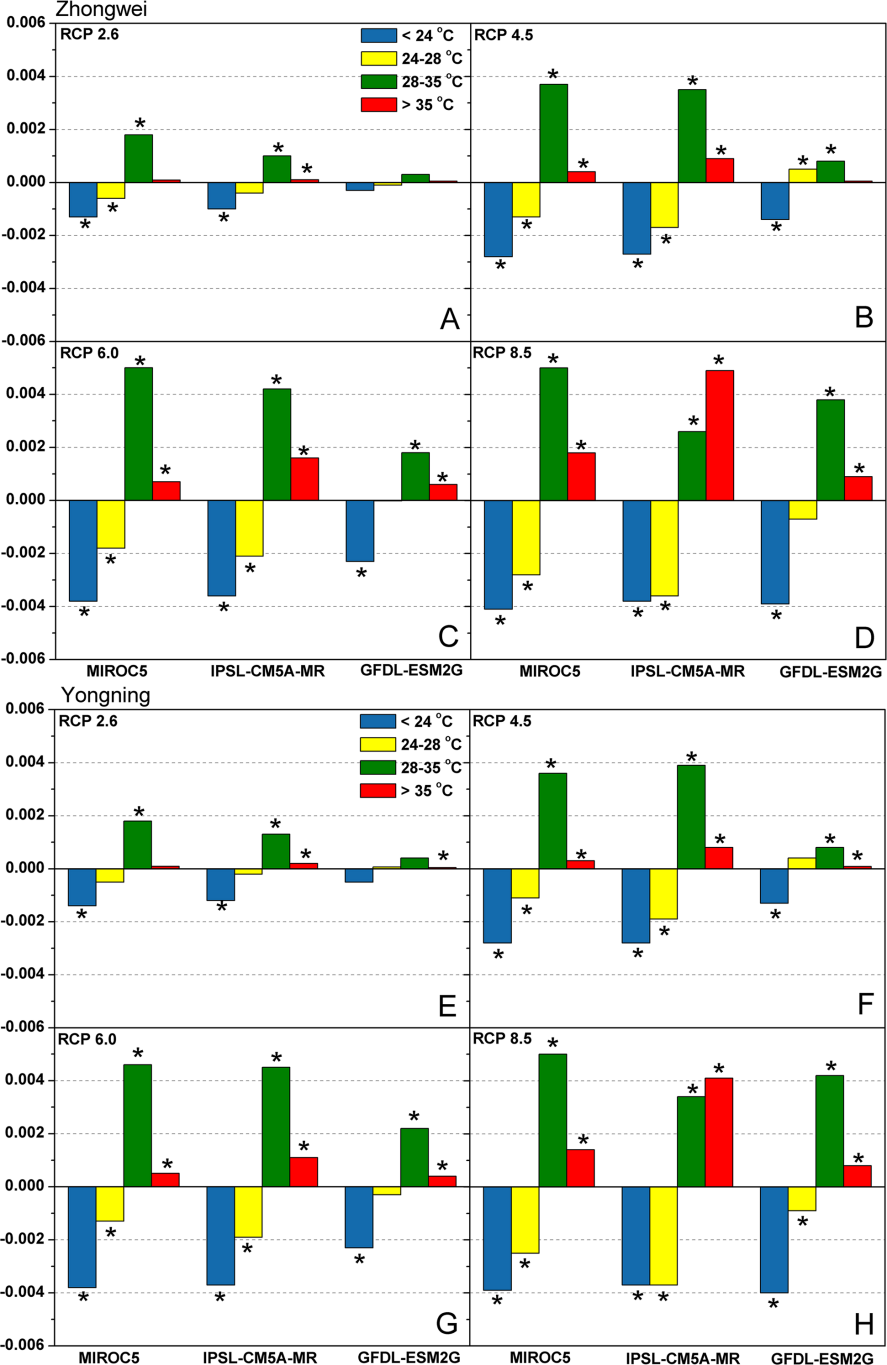
Trends of temperature frequency during the rice growth period over 2006–2100 for (A–D) the Zhongwei station and (E–H) the Yongning station. The asterisk (*) indicates that the trend passes the significance level test (*p* < 0.05).

Figure 6 shows the trends of LT, OT, HT, and EMT frequencies under all emission scenarios for the two study stations over the rice growth period from 2006 to 2100. Clearly, the frequencies of LT and OT decreased, while the frequencies of HT and EMT increased for most study cases. EMT in IPSL-CM5A-MR with RCP 8.5 had the most dramatic increase among all the cases. Such shifts indicated that higher temperatures became more dominant under future climate change scenarios and had a strong potential to affect rice processes.

### Impact of climate change on rice growth and development

#### Phenology

**Figure 7:**
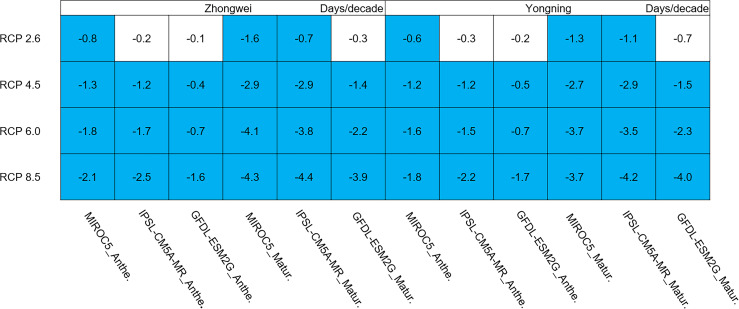
Trends of rice phonology for 2006–2100. The blue boxes show downward trends that pass the significance level test (*p* < 0.05), and the white boxes mean that this test is not passed.

Rice phenology was shortened significantly in all study cases, 87.5% of which passed the 95% significance test (Fig. 7; blue boxes). Since the growth period was almost directly proportional to the thermal time accumulation in the CERES-Rice model, the increased temperature could accelerate this accumulation, leading to earlier anthesis and maturity dates. For example, in the IPSL-CM5A-MR model with the fixed transplanting date (May 12), the anthesis date each year was 0.2, 1.2, 1.7, and 2.5 days/decade earlier for the Zhongwei station, and the maturity date each year was 0.7, 2.9, 3.8, and 4.4 days/decade earlier, corresponding to the RCP 2.6, RCP 4.5, RCP 6.0, and RCP 8.5 scenarios, respectively. The shorter phenology reduced the grain filling duration, which could result in a decreased rice yield.

#### Photosynthetic production

**Figure 8:**
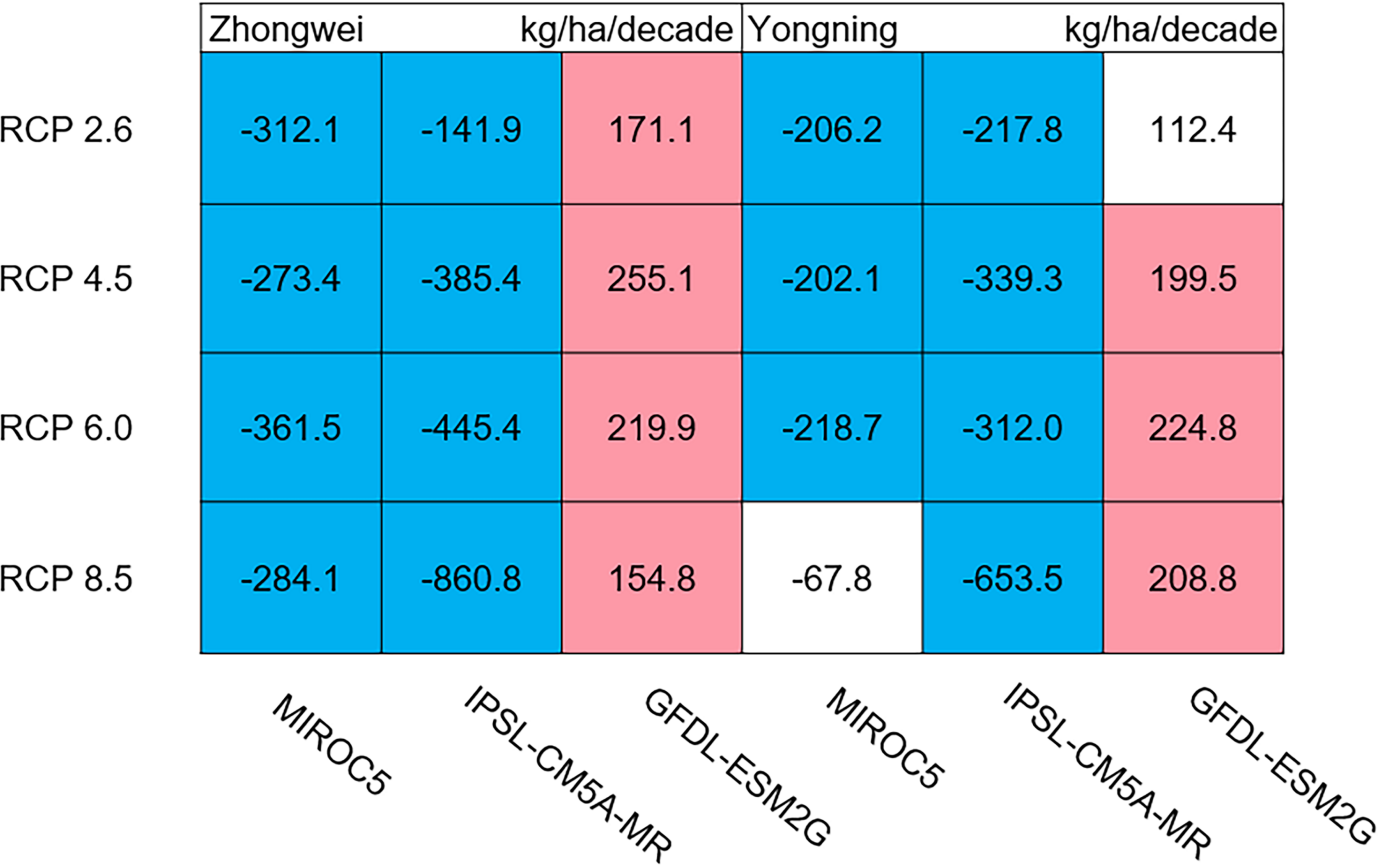
Trends of accumulated rice photosynthetic production for the entire rice growth period over 2006–2100. The colored boxes mean that the significance level test is passed (*p* < 0.05), and the white boxes mean that this test is not passed. Red and blue indicate the significant upward and downward trends, respectively.

In this study, the accumulated photosynthetic production for the entire rice growth period showed different trends under different climate change scenarios (Fig. 8). The IPSL-CM5A-MR and MIROC5 models generated negative trends for all emission scenarios, while the GFDL-ESM2G model produced positive trends. The majority of the trends (in blue and red) passed the 95% significance level test. As discussed previously, when the temperature was out of the optimal range, this reduced the photosynthetic production (Eq. (1)). Our simulations showed that the temperature shifted from the low and optimal ranges toward the high and extreme ones under almost all study scenarios (Fig. 6), and such shifts tended to reduce the photosynthetic production. Thus, it is clear that the negative trends of accumulated photosynthetic production were controlled by rising temperatures.

#### Aboveground biomass, leaf, stem, panicle, and yield

**Figure 9:**
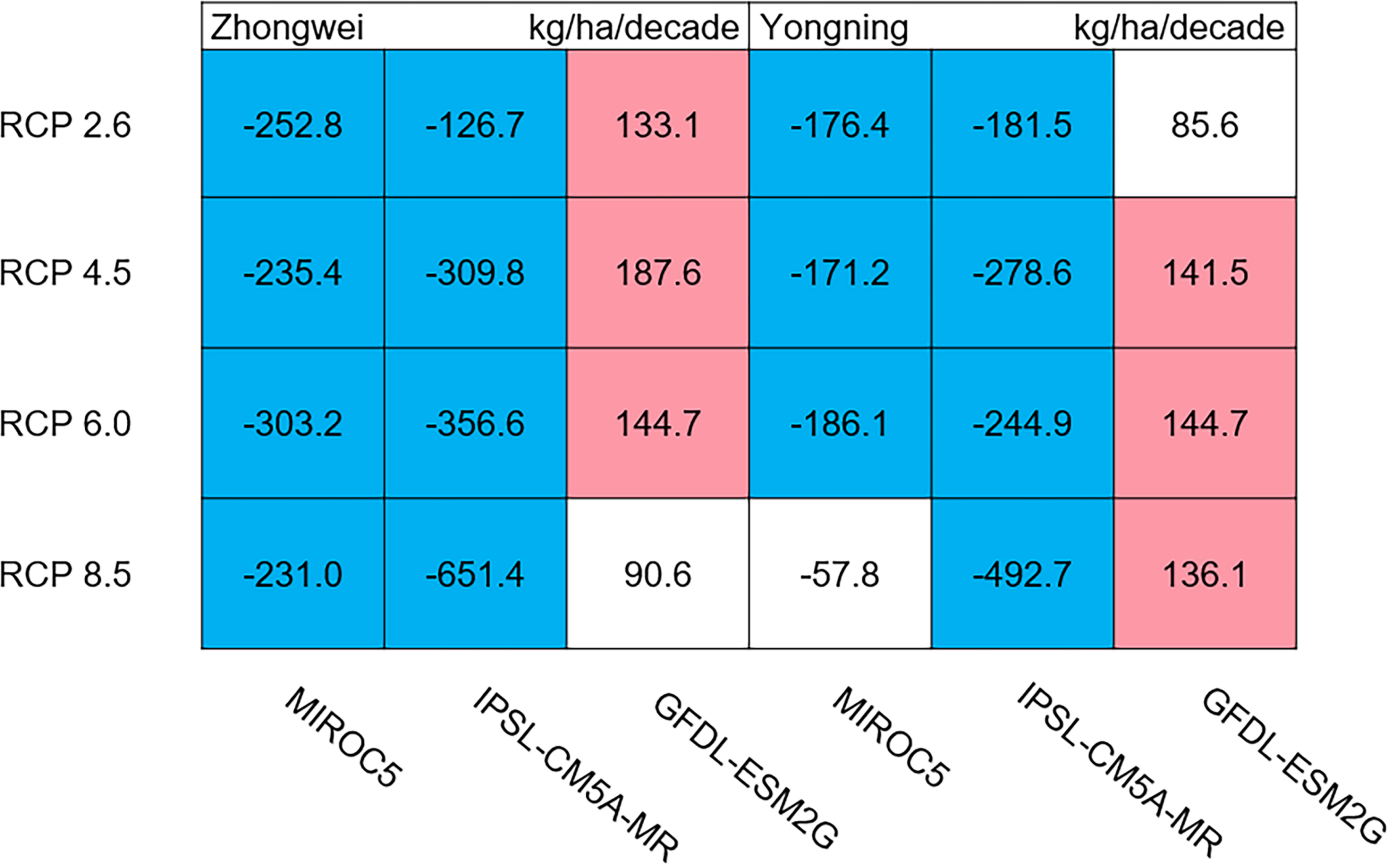
Trends of aboveground biomass at harvest for 2006–2100. The colored boxes mean that the significance level test is passed (*p* < 0.05), and the white boxes mean that this test is not passed. Red and blue indicate the significant upward and downward trends, respectively.

**Figure 10:**
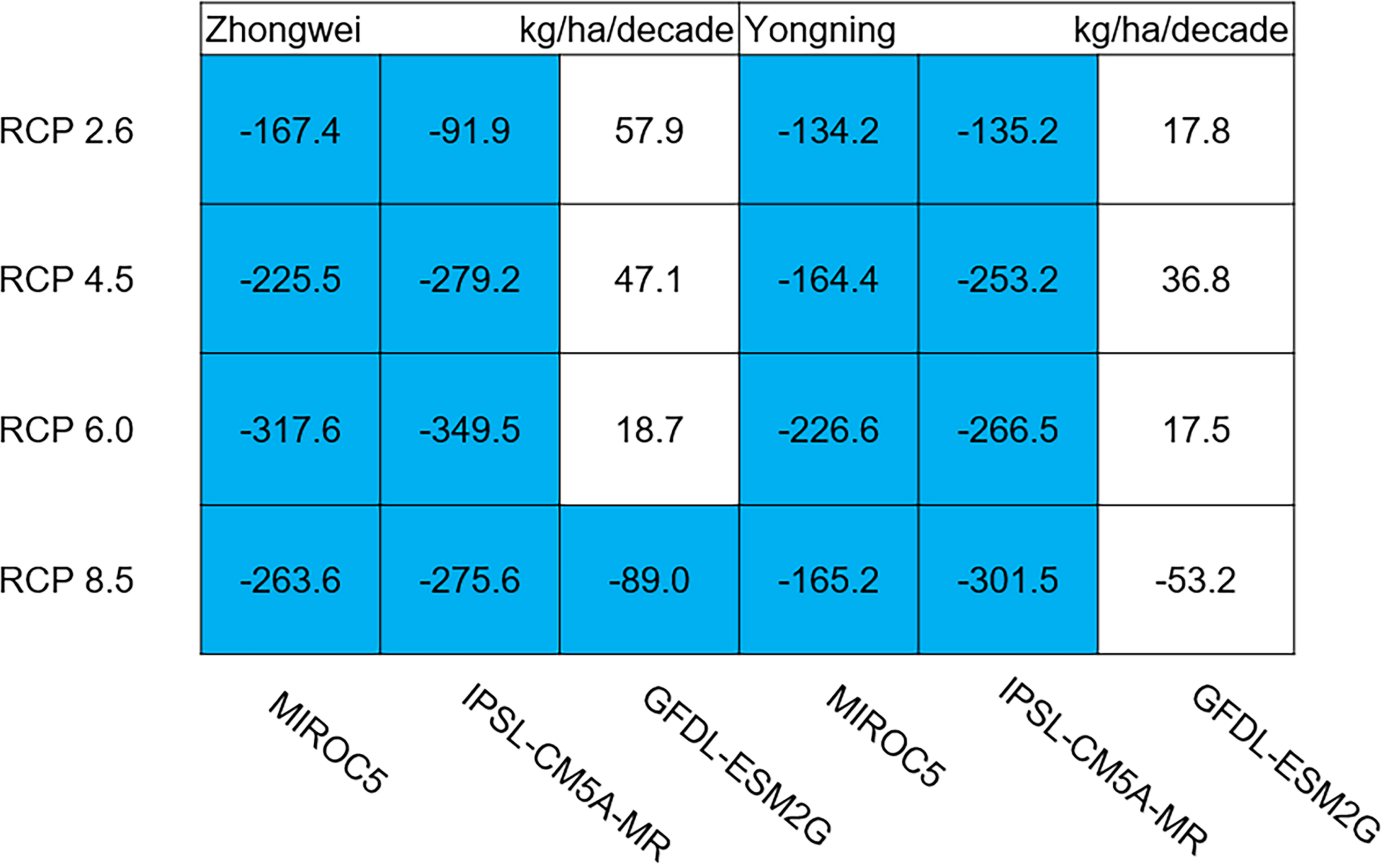
Trends of rice leaf and stem dry matter at harvest for 2006–2100. The blue boxes show downward trends that pass the significance level test (*p* < 0.05), and the white boxes mean that this test is not passed.

**Figure 11:**
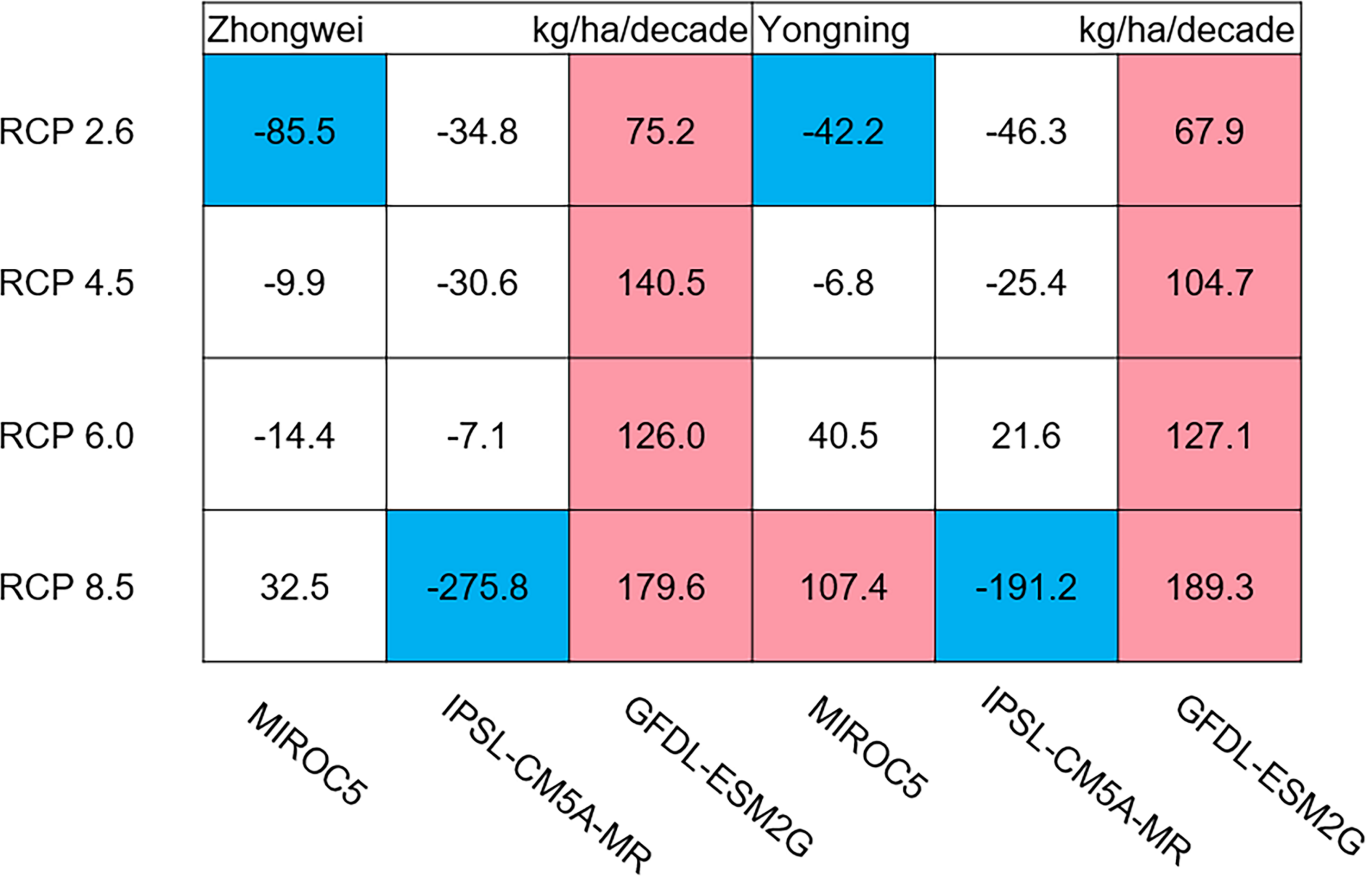
Trends of rice panicle dry matter at harvest for 2006–2100. The colored boxes mean that the significance level test is passed (*p* < 0.05), and the white boxes mean that this test is not passed. Red and blue indicate the significant upward and downward tren.

**Figure 12:**
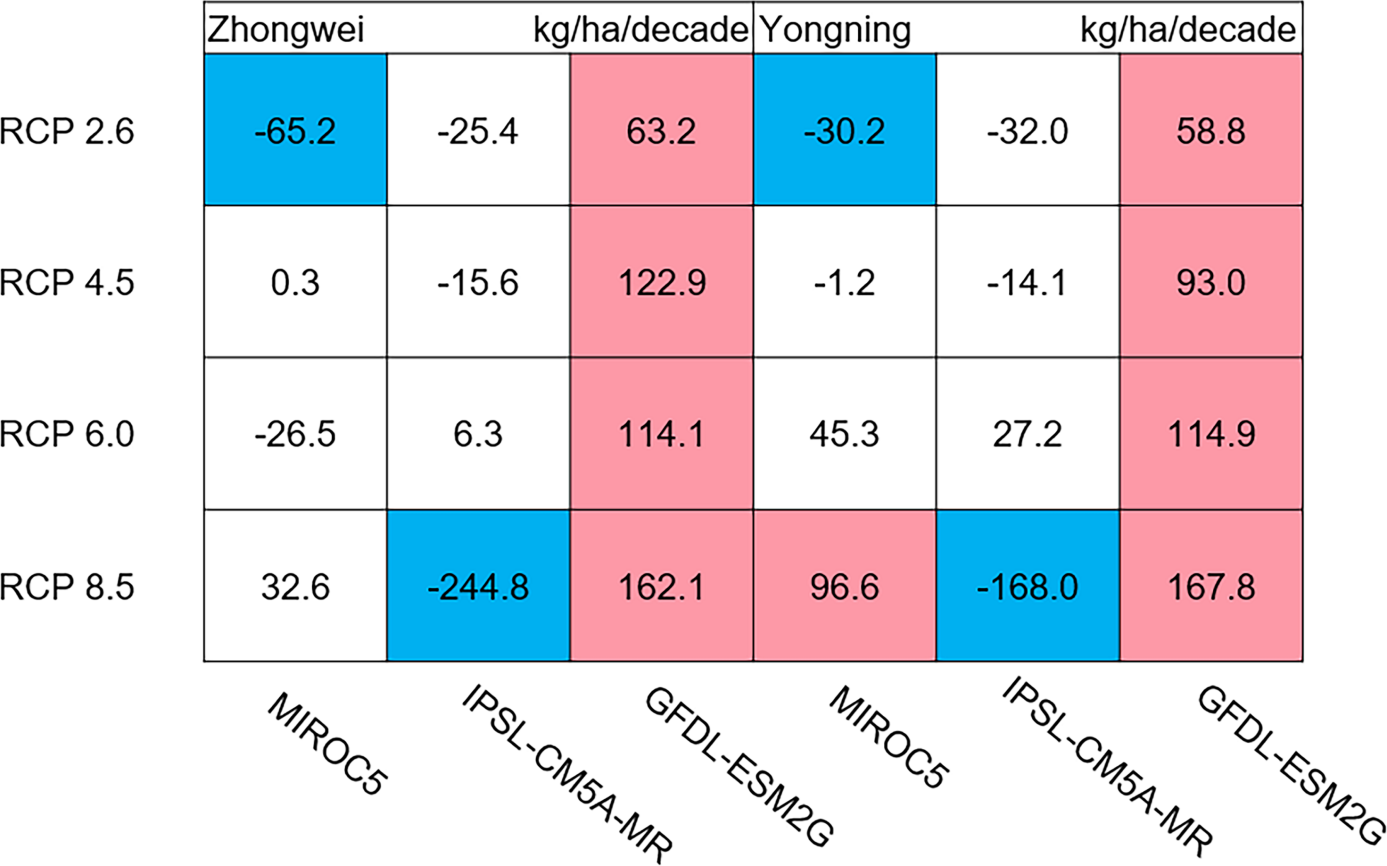
Trends of rice yield for 2006–2100. The colored boxes mean that the significance level test is passed (*p* < 0.05), and the white boxes mean that this test is not passed. Red and blue indicate the significant upward and downward trends, respectively.

The dry matter accumulation of rice organs was strongly affected by the photosynthetic intensity, whose long-term changes were very sensitive to the temperature frequency trends. Figure 9 shows that the trends of aboveground biomass at harvest were very similar to those of accumulated photosynthetic production, since the carbohydrates of the rice plant are produced by photosynthesis. The leaf and stem dry matter at harvest decreased significantly in the MIROC5 and IPSL-CM5A-MR models (*p* < 0.05; blue boxes in Fig. 10), while the trends of such dry matter mostly did not pass the 95% significance test in the GFDL-ESM2G model (white boxes in Fig. 10). The trends of panicle dry matter at harvest did not pass the 95% significance test for approximately 68.8% of the study cases in the MIROC5 and IPSL-CM5A-MR models (white boxes in Fig. 11). Two RCP 2.6 cases with MIROC5 and two RCP 8.5 cases with IPSL-CM5A-MR showed a significant downward trend of panicle dry matter, while an upward trend of this variable occurred for the MIROC5 with RCP 8.5 case with a *p*-value of less than 0.05, which was an exception. The panicle dry matter increased meaningfully for all study cases in the GFDL-ESM2G model (*p* < 0.05; red boxes in Fig. 11). Yield trends were very similar to those of the panicles (Fig. 12), because the panicle dry matter is mostly composed of grain weight (yield).

## Discussion

### Trends of temperature frequency

Based on above results, we can see that HT and EMT become dominant under future climate change with the decreased LT and OT frequencies (Fig. 6). Such shifts not only reduced rice photosynthesis (Eq. (1); Fig. 8), but limited fertilization as well (Fig. 11). Noticeably, with the daily maximum temperature over 35 °C for even a few hours, rice fertilization was restricted, severely reducing the yield, as verified by field experiments (Madan et al., 2012; Jagadish, Craufurd & Wheeler, 2007; Jagadish, Craufurd & Wheeler, 2008; Yoshida, Satake & Mackill, 1981; Satake & Yoshida, 1978). In the CERES-Rice model, this process was also considered. However, the model was run at a daily time step, which could cause errors in simulating rice fertilization.

### Photosynthetic production

In this study, the IPSL-CM5A-MR and MIROC5 models generated negative trends for all emission scenarios, while the GFDL-ESM2G model produced positive trends (Fig. 8), which need to be analyzed. As rice is a C_3_ plant, the elevated CO_2_ concentrations in the GCM projections enhance the activity of Rubisco, which accelerates the carboxylation of ribulose-1,5-bisphosphate (RuBP) and increases the net CO_2_ fixation rate, strengthening the photosynthetic production (Busch et al., 2013; Sasaki et al., 2007; Makino, 1994). Thus, the positive trends in the GFDL-ESM2G model indicate that the CO_2_ concentration can override the effects of rising temperature and increase photosynthetic production. Among the three GCMs, the GFDL-ESM2G model had the least temperature increase (Fig. 5), and the upward trends of the HT and EMT frequencies in this GCM were the slowest (Fig. 6). Thus, the CO_2_ concentration exerted a stronger effect on photosynthetic production than temperature in the GFDL-ESM2G model. On the other hand, rising temperature played a dominant role in affecting photosynthetic production than CO_2_ concentration in the other two GCMs. Different from the increased photosynthetic production in the field experiments (Wang et al., 2019a; Nam et al., 2013; Roy et al., 2012; Kim et al., 2011a; Cheng et al., 2010), the most scenarios in our studies showed a significant decrease in rice photosynthesis, which can be attributed to the random combinations of temperature and CO_2_ concentration in the field experiments.

### Dry matter of leaf, stem, panicle, and yield

The dry matter distribution among rice organs was affected by both photosynthesis and pollen fertilization. The rice photosynthesis in the field experiment mostly increased (Wang et al., 2019a; Nam et al., 2013; Roy et al., 2012; Kim et al., 2011a; Kim et al., 2011b; Cheng et al., 2010). These studies found that the dry matter of each organ increases with the increased CO_2_ concentration and temperature (Roy et al., 2012). On the other hand, the elevated temperature reduces the dry matter allocation to panicle due to the decreased spikelet numbers, significantly increasing the leaf and stem dry matter (Nam et al., 2013; Kim et al., 2011a; Kim et al., 2011b; Cheng et al., 2010). However, these results are based on the limited and random combinations of temperature and CO_2_ concentration. When photosynthesis is weakened, or when both photosynthesis and fertilization are restricted, the dry matter accumulation and distribution are uncertain.

Usually, leaf and stem dry matter is transferred to the panicles to form rice grains during the grain filling period (Stoy, 1980). When the leaf and stem dry matter decreased (Fig. 10), we did not see a significant decrease in the panicle dry matter except for two RCP 2.6 cases with MIROC5 and two RCP 8.5 cases with IPSL-CM5A-MR, where the panicle dry matter had a negative trend with a *p*-value of less than 0.05 (blue boxes). The trends of the projected fertilized spikelets (Fig. S1) were determined and were consistent with the changes in panicle dry matter in our CERES-Rice model projections. The insignificant changes in the fertilized spikelets and panicle dry matter imply that the demand and transfer of leaf and stem dry matter remained at a certain level, even if this dry matter decreased quite obviously due to the reduction in photosynthetic production. However, the panicle dry matter showed a distinct downward trend in the MIROC5 with RCP 2.6 cases and IPSL-CM5A-MR with RCP 8.5 cases. For the RCP 2.6 scenarios, the HT frequency in the MIROC5 model had the largest increase among the three models (Fig. 6), showing the largest photosynthetic production reduction (Fig. 8), and therefore decreasing the panicle dry matter much more than in the other two models. In these cases, the low carbon concentration with RCP 2.6 could not compensate for the large reduction in photosynthetic production and panicle dry matter caused by the increased HT frequency. In addition, the EMT frequency had the highest increase in the IPSL-CM5A-MR with the RCP 8.5 case among all the study cases, dramatically reducing the number of the fertilized spikelets and thus the panicle dry matter as shown in Fig. 11.

The rice panicle receives the photosynthetic product with a higher priority than the leaves and stem in the CERES-Rice model. The increase in panicle dry matter was clearly caused by the increased photosynthetic production in the GFDL-ESM2G model due to the more dominant effects of carbon over temperature (Figs. 10 and 11). This model generated an upward trend in the number of fertilized spikelets (Fig. S1) with the elevated photosynthetic production, indicating that the demand and transfer of the photosynthetic product to the panicles needed to hold a similar trend. This demand and transfer increase could reduce the percentage of photosynthetic product distribution to the leaves and stem and suppress their trends. To a very large extent, these processes led to insignificant changes in the leaves and stem (Fig. 10).

Yield trends were very similar to those of the panicles (Fig. 12), since changes in the panicles resulted directly in changes in the yield. Thus, the two variables shared similar trends behind their changes. The most dramatic yield reduction occurred in the IPSL-CM5A-MR with the RCP 8.5 case for the two study stations. As mentioned, the EMT frequency had the largest increase in this case. Previous studies indicated that EMT weakens the activity of pollen and inhibits anther dehiscence, resulting in poor pollen shed on the stigma (Prasad et al., 2006; Matsui, Omasa & Horie, 2000; Matsui, Omasa & Horie, 2001). The resulting processes severely weakened the rice fertilization and, thus, reduced the spikelet numbers and panicle dry matter. Finally, the highest EMT frequency led to the largest reduction in the rice yield.

### The effects of high temperature and CO_2_ on other rice type

The combined effects of increased temperature and CO_2_ concentration on the dry matter distribution in the organs of other rice types such as the indica rice may be different from those for japonica rice. The primary reason can be attributed to the higher heat resistance of indica rice than that of japonica rice (Wang et al., 2019b). Wang (2016) indicated that the spikelet degeneration rate of japonica rice cultivars increases more quickly than that of indica rice cultivars under high temperatures, implying that the indica rice may have a better heat adaptability. Such adaptability can affect the dry matter accumulation and distribution among the rice organs. Therefore, the indica rice may have a different response to the increased temperature and CO_2_ concentration, which needs to be further investigated.

### Limitations

Several limitations still exist in this study. Only two sites were selected for our simulations, and additional simulations need to be done in broader areas to draw more general conclusions. The single rice cultivar and fixed planting date are also possible shortcomings of this study. Different cultivars may have different sensitivities to high temperature, and the vegetative and reproductive growth phases usually vary with different cultivars. All these can affect the dry matter accumulation and distribution among the rice organs. Meanwhile, the effect of EMT on rice fertilization is likely avoided by adjusting the planting date, which need to be further tested. In addition, our simulations were conducted with sufficient water and fertilizer, which cannot always mimic the reality. These limitations need to be overcome in the future studies.

## Conclusion

In this study, we investigated the effects of temperature frequency trends on projected japonica rice yield and dry matter distribution with elevated carbon dioxide under future climate scenarios. We used the CERES-Rice model, which was quantitatively evaluated with historical field data for two representative stations, Zhongwei and Yongning, located in northwestern China. We selected the outputs from three GCMs—MIROC5, IPSL-CM5A-MR, and GFDL-ESM2G—under the RCP 2.6, 4.5, 6.0, and 8.5 scenarios, to drive the CERES-Rice model for future projections of rice growth and yield. The uncertainties in these outputs were reduced through statistical downscaling.

Our results showed that future temperature trends had the most significant impact on rice growth and development when compared with those of the other forcing variables, including precipitation and incoming solar radiation. In addition, the frequency of the higher than optimal temperature (∼26 °C) for rice growth showed a large increase in the future, which greatly restricted photosynthesis. The frequency of extreme temperatures (>35 °C) also increased, especially in IPSL-CM5A-MR with the RCP 8.5 case, which exerted a strong impact on rice fertilization and significantly reduced the yield. The increased temperature shortened the rice phenology and grain filling duration, potentially decreasing the yield. Meanwhile, the increased temperature suppressed the photosynthetic production, while the elevated CO_2_ tended to enhance this production, and the net result was determined by the dominant process. The trends of the aboveground biomass were downward when temperature became the major factor, and upward when CO_2_-fertilization dominated the photosynthetic production. The rice leaf and stem dry matter trends were affected not only by photosynthesis changes but also by the dry matter distribution to the panicles, the latter of which could significantly change those trends.

The rice panicle trends were closely related to the effects of temperature and CO_2_ on photosynthetic production, and how these trends changed depended on which factor played a major role. Extreme temperatures also remarkably affected panicle trends by reducing the number of fertilized spikelets. The rice yield shared the same trends with the panicle dry matter as panicle dry matter is mostly composed of grain weight (yield). These results provide a better understanding of improved japonica rice growth and yield projections under different climate change scenarios.

##  Supplemental Information

10.7717/peerj.11027/supp-1 Supplemental Information 1 Comparison between rice phenology and yield simulations and observations during 1997–2005

10.7717/peerj.11027/supp-2 Supplemental Information 2 The CRU temperature data and CMFD data link

10.7717/peerj.11027/supp-3 Supplemental Information 3 Downscaled data for Zhongwei station

10.7717/peerj.11027/supp-4 Supplemental Information 4 Downscaled data for Yongning station

10.7717/peerj.11027/supp-5 Supplemental Information 5 Projected rice phenology and yield

10.7717/peerj.11027/supp-6 Supplemental Information 6 Projected rice organ dry matter and photosynthetic production

10.7717/peerj.11027/supp-7 Supplemental Information 7 The same as Fig. 9, but for rice projected fertilized spikelets

## Additional Information and Declarations

### Data Availability

The following information was supplied regarding data availability: Raw data is available in the Supplemental Files.
