# Supplementary material for: Effects of temperature frequency trends on projected japonica rice (Oryza sativa L.) yield and dry matter distribution with elevated carbon dioxide
Source: PeerJ. 2021 Mar 11;9:e11027. doi: 10.7717/peerj.11027 (PMC7956007; doi:10.7717/peerj.11027)
Supplement: Supplemental Information 2 [file peerj-09-11027-s002.docx]

CRU temperature data link:

<https://svn-ccsm-inputdata.cgd.ucar.edu/trunk/inputdata/atm/datm7/atm_forcing.datm7.cruncep_qianFill.0.5d.v7.c160715/TPHWL6Hrly/>

CMDF data link:

https://data.tpdc.ac.cn/zh-hans/data/8028b944-daaa-4511-8769-965612652c49/
