# Supplementary figures and images for: Effects of temperature frequency trends on projected japonica rice (Oryza sativa L.) yield and dry matter distribution with elevated carbon dioxide
Source: PeerJ. 2021 Mar 11;9:e11027. doi: 10.7717/peerj.11027 (PMC7956007; doi:10.7717/peerj.11027)

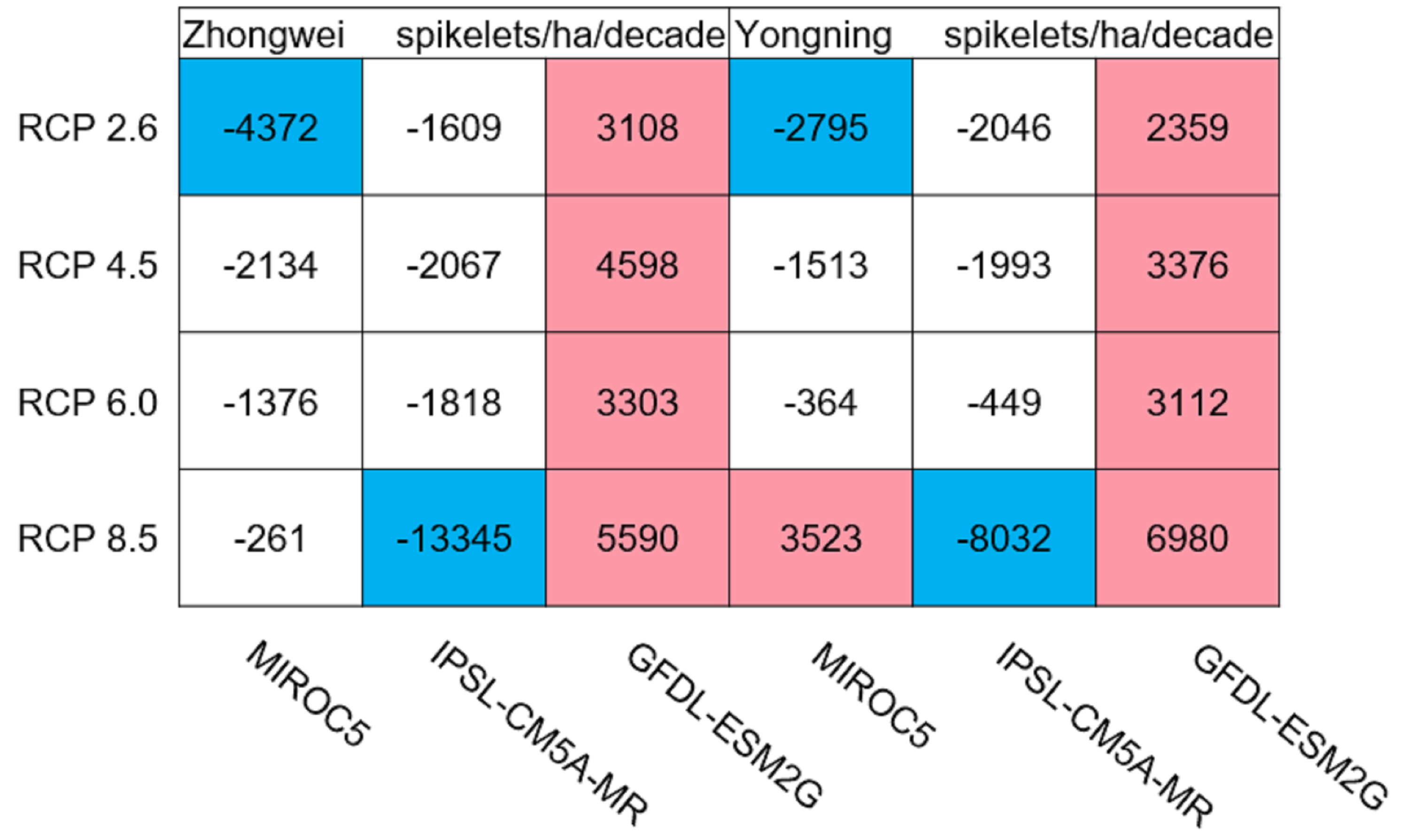

Supplement: Supplemental Information 7 [file peerj-09-11027-s007.png]
